# 1,4-Bis(methyl­sulfan­yl)naphthalene

**DOI:** 10.1107/S1600536809018650

**Published:** 2009-05-23

**Authors:** Ísmail Çelik, Mehmet Akkurt, Ayşegül Şenocak, Osman Çakmak, Laura Torre-Fernández, Santiago García-Granda

**Affiliations:** a Department of Physics, Faculty of Arts and Sciences, Cumhuriyet University, 06532 Sivas, Turkey; b Department of Physics, Faculty of Arts and Sciences, Erciyes University, 38039 Kayseri, Turkey; c Department of Chemistry, Faculty of Arts and Sciences, Gaziosmanpaşa University, 60240 Tokat, Turkey; d Departamento Química Física y Analítica, Facultad de Química, Universidad Oviedo, C/ Julián Clavería, 8, 33006 Oviedo (Asturias), Spain

## Abstract

The mol­ecule of the title compound, C_12_H_12_S_2_, is close to planar, with the methyl C atoms deviating by 0.019 (1) and 0.221 (2) Å from the naphthalene mean plane. In the crystal structure, the shortest S⋯S contact of 3.6864 (9) Å is longer than the van der Waals contact distance.

## Related literature

For general background, see: Underhill (1992 ▶); Öncü *et al.* (2006 ▶). For related structures, see: Noreland *et al.* (1992 ▶, 1993 ▶). For bond-length data, see: Allen *et al.* (1987 ▶). For van der Waals radii, see: Bondi (1964 ▶).
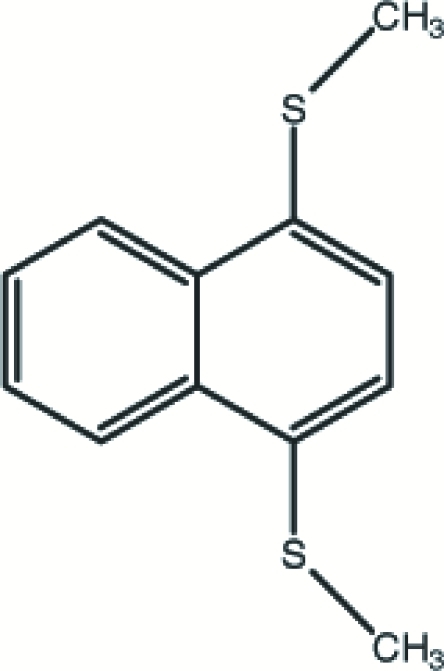


## Experimental

### 

#### Crystal data

C_12_H_12_S_2_
*M* *_r_* = 220.34
Monoclinic, 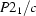

*a* = 15.203 (3) Å
*b* = 10.246 (2) Å
*c* = 7.1750 (14) Å
β = 99.43 (3)°
*V* = 1102.6 (4) Å^3^
*Z* = 4
Synchrotron radiation
λ = 0.75140 Å
μ = 0.44 mm^−1^
*T* = 153 K
0.11 × 0.08 × 0.02 mm

#### Data collection

Bruker *P*4 diffractometer
Absorption correction: part of the refinement model (Δ*F*) (*XABS2*; Parkin *et al.*, 1995 ▶) *T*_min_ = 0.832, *T*_max_ = 0.991
5197 measured reflections
3013 independent reflections
2619 reflections with *I* > 2σ(*I*)
*R*_int_ = 0.019

#### Refinement

*R*[*F* ^2^ > 2σ(*F* ^2^)] = 0.036
*wR*(*F* ^2^) = 0.109
*S* = 1.07
3013 reflections
127 parameters
H-atom parameters constrained
Δρ_max_ = 0.25 e Å^−3^
Δρ_min_ = −0.25 e Å^−3^

### 

Data collection: *XSCANS* (Bruker, 1996 ▶); cell refinement: *SCALEPACK* (Otwinowski & Minor, 1997 ▶); data reduction: *DENZO* (Otwinowski & Minor, 1997 ▶) and *SCALEPACK*; program(s) used to solve structure: direct methods using *SIR2004* (Burla *et al.*, 2005 ▶); program(s) used to refine structure: *SHELXL97* (Sheldrick, 2008 ▶); molecular graphics: *ORTEP-3 for Windows* (Farrugia, 1997 ▶); software used to prepare material for publication: *WinGX* (Farrugia, 1999 ▶).

## Supplementary Material

Crystal structure: contains datablocks global, I. DOI: 10.1107/S1600536809018650/hb2962sup1.cif

Structure factors: contains datablocks I. DOI: 10.1107/S1600536809018650/hb2962Isup2.hkl

Additional supplementary materials:  crystallographic information; 3D view; checkCIF report

## Figures and Tables

**Fig. 1.**
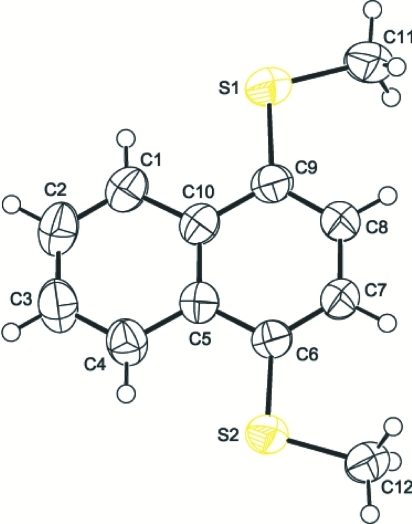
The molecular structure of (I), with displacement ellipsoids drawn at the 50% probability level.

**Fig. 2.**
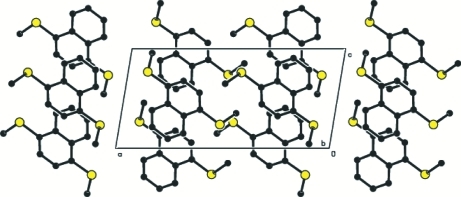
View of the molecular packing diagram of compound (I) viewed down b axis. H atoms have been omitted for clarity.

## supplementary crystallographic information

### Comment

Molecular electronics have attracted considerable attention because they are used for production electronic switches, memory cells or sensors. The most extensive range of the set compounds is based on planar organic donor molecules containing S or Se atoms (*e.g.* Underhill, 1992; Noreland *et al.*, 1992; Noreland *et al.*, 1993; Öncü *et al.*, 2006. In this study, the synthesis and structure of the title compound, (I), are reported.

As shown in Fig. 1, the naphthalene group (C1–C10) of the title molecule (I) is essentially planar, with maximum deviations of -0.014 (1), -0.014 (1) and 0.018 (1) Å for C4, C8 and C6, respectively. Deviations from planarity in the title molecule which were calculated to the least-squares plane of the naphthalene group are: 0.014 (1) Å for S1, 0.112 (1) Å for S2, -0.221 (2) Å for C11 and -0.019 (1) Å for C12. The bond lengths and angles in (I) show normal values (Allen *et al.*, 1987). The molecular packing of (I) as seen down the *a* axis is illustrated in Fig. 2. There is only one S···S contact near the van der Waals contact distance, 3.6 Å (Bondi, 1964): S2···S2^i^ 3.6864 (9) Å [symmetry code: (i) -*x* + 1, -*y*, -*z*].

### Experimental

The complete reaction was carried out under nitrogen atmosphere and 195 K. t-BuLi (10 ml, 14 mmol) solution was added to 1,4-dibromonaphthalene (1 g, 3.5 mmol) solved in dry THF (12 ml) with continuous stirring. Meanwhile, red colour was observed because of LiBr formation. Later, to the resulting reaction mixture was added dropwise (CH_3_S)_2 _(1.24 ml, 14 mmol). After 1 h, the reaction was ended. Reaction mixture warming to room temperature was extracted with Et_2_O (3 × 25 ml). The extract was dried over Na_2_SO_4_ and concentrated. 1,4-Bis(methylthio)naphthalene as white needle-like crystals was obtained by crystallization from crude product solved in dichloromethane:hexan (1:19) system (708 mg, 92%), m.p. 369–371 K (lit. 370–371 K).

### Refinement

H atoms were positioned geometrically and allowed to ride on their parent atoms, with C—H = 0.93 Å and *U*_iso_(H) = 1.2*U*_eq_(C) for aromatic H atoms, and 0.9 Å and *U*_iso_(H) = 1.5*U*_eq_(C) for methyl H atoms.

### Crystal data

**Table:**

C_12_H_12_S_2_	*F*(000) = 464
*M**_r_* = 220.34	*D*_x_ = 1.327 Mg m^−^^3^
Monoclinic, *P*2_1_/*c*	Synchrotron radiation, λ = 0.75140 Å
Hall symbol: -P 2ybc	Cell parameters from 40396 reflections
*a* = 15.203 (3) Å	θ = 1.0–27.0°
*b* = 10.246 (2) Å	µ = 0.44 mm^−^^1^
*c* = 7.1750 (14) Å	*T* = 153 K
β = 99.43 (3)°	Prism, colourless
*V* = 1102.6 (4) Å^3^	0.11 × 0.08 × 0.02 mm
*Z* = 4	

### Data collection

**Table:**

Bruker P4 diffractometer	3013 independent reflections
Radiation source: sealed tube	2619 reflections with *I* > 2σ(*I*)
graphite	*R*_int_ = 0.019
ω scans	θ_max_ = 33.8°, θ_min_ = 2.6°
Absorption correction: part of the refinement model (Δ*F*) (*XABS2*; Parkin *et al.*, 1995)	*h* = −20→20
*T*_min_ = 0.832, *T*_max_ = 0.991	*k* = −14→14
5197 measured reflections	*l* = −9→0

### Refinement

**Table:**

Refinement on *F*^2^	Primary atom site location: structure-invariant direct methods
Least-squares matrix: full	Secondary atom site location: difference Fourier map
*R*[*F*^2^ > 2σ(*F*^2^)] = 0.036	Hydrogen site location: inferred from neighbouring sites
*wR*(*F*^2^) = 0.109	H-atom parameters constrained
*S* = 1.07	*w* = 1/[Σ^2^(*F*_o_^2^) + (0.0553*P*)^2^ + 0.1466*P*] where *P* = (*F*_o_^2^ + 2*F*_c_^2^)/3
3013 reflections	(Δ/σ)_max_ = 0.001
127 parameters	Δρ_max_ = 0.25 e Å^−^^3^
0 restraints	Δρ_min_ = −0.25 e Å^−^^3^

### Special details

**Table:**

Experimental. Cubic fit to sin(theta)/lambda - 24 parameters
Geometry. Bond distances, angles *etc*. have been calculated using the rounded fractional coordinates. All su's are estimated from the variances of the (full) variance-covariance matrix. The cell e.s.d.'s are taken into account in the estimation of distances, angles and torsion angles
Refinement. Refinement on *F*^2^ for ALL reflections except those flagged by the user for potential systematic errors. Weighted *R*-factors *wR* and all goodnesses of fit *S* are based on *F*^2^, conventional *R*-factors *R* are based on *F*, with *F* set to zero for negative *F*^2^. The observed criterion of *F*^2^ > σ(*F*^2^) is used only for calculating -*R*-factor-obs *etc*. and is not relevant to the choice of reflections for refinement. *R*-factors based on *F*^2^ are statistically about twice as large as those based on *F*, and *R*-factors based on ALL data will be even larger.

### Fractional atomic coordinates and isotropic or equivalent isotropic displacement parameters (Å^2^)

**Table:**

	*x*	*y*	*z*	*U*_iso_*/*U*_eq_	
S1	0.90863 (2)	−0.04680 (4)	0.76728 (6)	0.0671 (2)	
S2	0.53023 (2)	−0.06992 (3)	0.23860 (5)	0.0558 (1)	
C1	0.85913 (9)	0.07840 (13)	0.3828 (2)	0.0578 (4)	
C2	0.84525 (11)	0.14096 (16)	0.2131 (2)	0.0700 (5)	
C3	0.76299 (11)	0.13355 (17)	0.0952 (2)	0.0714 (5)	
C4	0.69472 (10)	0.06351 (14)	0.1496 (2)	0.0592 (4)	
C5	0.70573 (8)	−0.00085 (11)	0.32680 (17)	0.0468 (3)	
C6	0.63478 (8)	−0.07293 (11)	0.38797 (18)	0.0469 (3)	
C7	0.65018 (9)	−0.13631 (14)	0.55763 (19)	0.0562 (4)	
C8	0.73388 (9)	−0.13004 (14)	0.67599 (19)	0.0585 (4)	
C9	0.80296 (8)	−0.05983 (12)	0.62517 (19)	0.0511 (4)	
C10	0.79008 (8)	0.00626 (11)	0.44685 (18)	0.0478 (3)	
C11	0.90238 (12)	−0.15800 (18)	0.9562 (3)	0.0818 (6)	
C12	0.46292 (9)	−0.17829 (14)	0.3515 (2)	0.0625 (4)	
H1	0.91490	0.08300	0.45830	0.0690*	
H2	0.89120	0.18910	0.17560	0.0840*	
H3	0.75440	0.17620	−0.02070	0.0860*	
H4	0.64030	0.05810	0.06910	0.0710*	
H7	0.60450	−0.18460	0.59600	0.0670*	
H8	0.74230	−0.17440	0.79060	0.0700*	
H11A	0.95780	−0.15720	1.04240	0.1230*	
H11B	0.89100	−0.24440	0.90620	0.1230*	
H11C	0.85490	−0.13220	1.02170	0.1230*	
H12A	0.40410	−0.18270	0.27870	0.0940*	
H12B	0.45940	−0.14650	0.47600	0.0940*	
H12C	0.48920	−0.26370	0.36040	0.0940*	

### Atomic displacement parameters (Å^2^)

**Table:**

	*U*^11^	*U*^22^	*U*^33^	*U*^12^	*U*^13^	*U*^23^
S1	0.0534 (2)	0.0744 (3)	0.0682 (3)	−0.0142 (2)	−0.0060 (2)	0.0043 (2)
S2	0.0519 (2)	0.0567 (2)	0.0553 (2)	−0.0028 (1)	−0.0020 (1)	0.0051 (1)
C1	0.0503 (7)	0.0558 (7)	0.0691 (8)	−0.0030 (5)	0.0149 (6)	0.0008 (6)
C2	0.0643 (8)	0.0693 (9)	0.0818 (10)	−0.0055 (7)	0.0280 (7)	0.0134 (7)
C3	0.0707 (9)	0.0773 (10)	0.0696 (9)	0.0035 (7)	0.0212 (7)	0.0236 (8)
C4	0.0578 (7)	0.0612 (7)	0.0593 (8)	0.0062 (5)	0.0117 (6)	0.0111 (6)
C5	0.0494 (6)	0.0417 (5)	0.0504 (6)	0.0037 (4)	0.0116 (4)	0.0001 (4)
C6	0.0453 (6)	0.0447 (5)	0.0497 (6)	0.0001 (4)	0.0047 (4)	−0.0012 (4)
C7	0.0490 (6)	0.0610 (7)	0.0571 (7)	−0.0094 (5)	0.0043 (5)	0.0098 (6)
C8	0.0540 (7)	0.0660 (8)	0.0528 (7)	−0.0088 (6)	0.0007 (5)	0.0117 (6)
C9	0.0473 (6)	0.0505 (6)	0.0537 (7)	−0.0036 (5)	0.0031 (5)	−0.0019 (5)
C10	0.0484 (6)	0.0411 (5)	0.0550 (6)	0.0002 (4)	0.0115 (5)	−0.0026 (5)
C11	0.0770 (10)	0.0848 (11)	0.0728 (10)	−0.0151 (9)	−0.0198 (8)	0.0164 (8)
C12	0.0536 (7)	0.0643 (8)	0.0667 (8)	−0.0097 (6)	0.0017 (6)	0.0023 (6)

### Geometric parameters (Å, °)

**Table:**

S1—C9	1.7611 (14)	C9—C10	1.4324 (18)
S1—C11	1.785 (2)	C1—H1	0.9300
S2—C6	1.7657 (13)	C2—H2	0.9300
S2—C12	1.7911 (15)	C3—H3	0.9300
C1—C2	1.362 (2)	C4—H4	0.9300
C1—C10	1.4204 (19)	C7—H7	0.9300
C2—C3	1.392 (2)	C8—H8	0.9300
C3—C4	1.370 (2)	C11—H11A	0.9600
C4—C5	1.4176 (19)	C11—H11B	0.9600
C5—C6	1.4341 (17)	C11—H11C	0.9600
C5—C10	1.4247 (18)	C12—H12A	0.9600
C6—C7	1.3656 (19)	C12—H12B	0.9600
C7—C8	1.411 (2)	C12—H12C	0.9600
C8—C9	1.3707 (19)		
			
S2···S2^i^	3.6864 (9)		
			
C9—S1—C11	103.55 (8)	C1—C2—H2	120.00
C6—S2—C12	103.82 (7)	C3—C2—H2	120.00
C2—C1—C10	121.32 (14)	C2—C3—H3	120.00
C1—C2—C3	120.65 (15)	C4—C3—H3	120.00
C2—C3—C4	120.16 (14)	C3—C4—H4	120.00
C3—C4—C5	121.03 (14)	C5—C4—H4	119.00
C4—C5—C6	121.99 (12)	C6—C7—H7	119.00
C4—C5—C10	118.74 (12)	C8—C7—H7	119.00
C6—C5—C10	119.27 (11)	C7—C8—H8	119.00
S2—C6—C5	116.91 (10)	C9—C8—H8	119.00
S2—C6—C7	123.72 (10)	S1—C11—H11A	109.00
C5—C6—C7	119.34 (12)	S1—C11—H11B	109.00
C6—C7—C8	121.39 (13)	S1—C11—H11C	109.00
C7—C8—C9	121.24 (13)	H11A—C11—H11B	110.00
S1—C9—C8	123.49 (11)	H11A—C11—H11C	109.00
S1—C9—C10	117.36 (10)	H11B—C11—H11C	109.00
C8—C9—C10	119.15 (12)	S2—C12—H12A	109.00
C1—C10—C5	118.08 (12)	S2—C12—H12B	109.00
C1—C10—C9	122.34 (12)	S2—C12—H12C	109.00
C5—C10—C9	119.58 (11)	H12A—C12—H12B	109.00
C2—C1—H1	119.00	H12A—C12—H12C	109.00
C10—C1—H1	119.00	H12B—C12—H12C	109.00
			
C11—S1—C9—C8	7.08 (14)	C10—C5—C6—C7	−1.81 (18)
C11—S1—C9—C10	−172.38 (11)	C4—C5—C10—C1	0.28 (18)
C12—S2—C6—C5	175.91 (10)	C4—C5—C10—C9	−179.02 (12)
C12—S2—C6—C7	−5.94 (13)	C6—C5—C10—C1	−179.94 (12)
C10—C1—C2—C3	−1.4 (2)	C6—C5—C10—C9	0.76 (17)
C2—C1—C10—C5	1.0 (2)	S2—C6—C7—C8	−176.70 (11)
C2—C1—C10—C9	−179.70 (14)	C5—C6—C7—C8	1.4 (2)
C1—C2—C3—C4	0.4 (2)	C6—C7—C8—C9	0.1 (2)
C2—C3—C4—C5	0.9 (2)	C7—C8—C9—S1	179.38 (11)
C3—C4—C5—C6	178.97 (14)	C7—C8—C9—C10	−1.2 (2)
C3—C4—C5—C10	−1.3 (2)	S1—C9—C10—C1	0.93 (17)
C4—C5—C6—S2	−3.80 (16)	S1—C9—C10—C5	−179.80 (10)
C4—C5—C6—C7	177.96 (13)	C8—C9—C10—C1	−178.56 (13)
C10—C5—C6—S2	176.43 (9)	C8—C9—C10—C5	0.71 (18)

Symmetry codes: (i) −*x*+1, −*y*, −*z*.

### Hydrogen-bond geometry (Å, °)

**Table:**

*D*—H···*A*	*D*—H	H···*A*	*D*···*A*	*D*—H···*A*
C1—H1···S1	0.93	2.60	3.0235 (16)	108
C4—H4···S2	0.93	2.58	3.0090 (17)	108
